# Inadequate description of educational interventions in ongoing randomized controlled trials

**DOI:** 10.1186/1745-6215-13-63

**Published:** 2012-05-18

**Authors:** Cécile Pino, Isabelle Boutron, Philippe Ravaud

**Affiliations:** 1 INSERM, U738, Paris, France; 2 AP-HP (Assistance Publique des Hôpitaux de Paris), Hôpital Hôtel Dieu, Centre d’ Epidémiologie Clinique, Paris, France; 3 Université Paris Descartes, Faculté de Médicine, Paris, France

## Abstract

**Background:**

The registration of clinical trials has been promoted to prevent publication bias and increase research transparency. Despite general agreement about the minimum amount of information needed for trial registration, we lack clear guidance on descriptions of non-pharmacologic interventions in trial registries. We aimed to evaluate the quality of registry descriptions of non-pharmacologic interventions assessed in ongoing randomized controlled trials (RCTs) of patient education.

**Methods:**

On 6 May 2009, we searched for all ongoing RCTs registered in the 10 trial registries accessible through the World Health Organization International Clinical Trials Registry Platform. We included trials evaluating an educational intervention (that is, designed to teach or train patients about their own health) and dedicated to participants, their family members or home caregivers. We used a standardized data extraction form to collect data related to the description of the experimental intervention, the centers, and the caregivers.

**Results:**

We selected 268 of 642 potentially eligible studies and appraised a random sample of 150 records. All selected trials were registered in 4 registers, mainly ClinicalTrials.gov (61%). The median [interquartile range] target sample size was 205 [100 to 400] patients. The comparator was mainly usual care (47%) or active treatment (47%). A minority of records (17%, 95% CI 11 to 23%) reported an overall adequate description of the intervention (that is, description that reported the content, mode of delivery, number, frequency, duration of sessions and overall duration of the intervention). Further, for most reports (59%), important information about the content of the intervention was missing. The description of the mode of delivery of the intervention was reported for 52% of studies, the number of sessions for 74%, the frequency of sessions for 58%, the duration of each session for 45% and the overall duration for 63%. Information about the caregivers was missing for 70% of trials. Most trials (73%) took place in the United States or United Kingdom, 64% involved only one centre, and participating centers were mainly tertiary-care, academic or university hospitals (51%).

**Conclusions:**

Educational interventions assessed in ongoing RCTs of educational interventions are poorly described in trial registries. The lack of adequate description raises doubts about the ability of trial registration to help patients and researchers know about the treatment evaluated in trials of education.

## Background

The International Committee of Medical Journal Editors (ICMJE) has promoted the registration of clinical trials to meet the challenge of research transparency, including the ability to adequately address publication bias and selective reporting, and reduce the amount of wasted research [1-4]. Since 2005, the ICMJE has required registration of all clinical trials as a condition of consideration for publication [1].

At present, agreement exists on the minimum protocol information that should be registered for a trial, that is, the 20-item World Health Organization (WHO) Trial Registration Data Set [5],[6]. One item of this dataset is devoted to the intervention. However, recommendations for the reporting of this item focus on pharmacologic treatments. No clear guidance is provided for descriptions of non-pharmacologic interventions in trial registries.

Nevertheless, reporting non-pharmacologic treatments is important but difficult. Non-pharmacologic interventions, such as educational interventions, are usually complex interventions involving several components [7] and so are difficult to describe, standardize, reproduce and administer consistently to all patients [8]. In addition, these interventions usually strongly depend on the context of care, such as the care provider’s expertise [9].

The frequency of noncommunicable diseases is increasing, and determining ways to support people with chronic illness is a strong focus of healthcare agendas [10]. Patient education through various educational interventions plays an essential role for adequately understanding and managing chronic diseases [11]. Thus, results from a growing number of randomized controlled trials (RCTs) assessing educational interventions have been published in the last 10 years and currently represent about 20% of the published results of RCTs [12].

We aimed to evaluate the quality of descriptions of interventions assessed in RCTs registered in the WHO International Clinical Trials Registry Platform (ICTRP). We focused on descriptions of the intervention and the context of care in the field of patient education.

## Methods

### Search strategy

On 6 May 2009, we searched for all ongoing RCTs assessing an educational intervention that were registered in the 10 registries accessible by the WHO search portal [13]: Australian New Zealand Clinical Trials Registry, Chinese Clinical Trial Register, ClinicalTrials.gov, Clinical Trials Registry – India, German Clinical Trials Register, Iranian Registry of Clinical Trials, ISRCTN.org, Sri Lanka Clinical Trials Registry, The Netherlands National Trial Register and EU Clinical Trials Register. We used this platform because it allows access to all primary registries meeting the WHO criteria. We searched for the intervention “education” for ongoing trials currently recruiting subjects.

### Eligibility criteria

Eligibility criteria were an ongoing randomized clinical trial (recorded as “randomized” or described as randomized in the description of the intervention), evaluating an educational intervention (that is, designed to teach or train patients concerning their own health [11]) and dedicated to participants (healthy or sick), their family members or home caregivers. We excluded interventions involving health workers if the intervention was integrally delivered to them and curative interventions designed to treat mental disorders, such as psychotherapy.

Two reviewers independently screened all potentially eligible studies. All disagreements were resolved by consensus and with a third reviewer if necessary.

From the selected records, we randomly chose a sample of 150 records (50%).

### Data collection

We developed a data extraction form and two reviewers (CP, IB) independently tested it with a sample of 20 studies. The agreement rate between the reviewers ranged from 0.7 to 1.

Then, a single reviewer (CP) used the standardized data extraction form to collect data from 1) the record in the WHO International Clinical Trials Registry Platform, 2) the record in the primary registry (for example, ClinicalTrials.gov, Netherlands National Trial, Australian New Zealand Clinical Trials Registry (…)) and 3) the website included in the record or previous publications listed in the record. All materials mentioned were searched. We did not search the Internet or write to the investigators for additional data. We collected the following data.

### General characteristics

We recorded the primary registry, date of registration, study design, comparator, sample size, number of arms and number of experimental arms. We also noted the type of patients enrolled (healthy or sick), mode of recruitment, age of study participants, and centres involved (country, number of centers, type of center).

To determine the number of participating centers, we used the number of centers reported, if any, in the general description of the trial or the number of centers mentioned in the WHO record; or in the primary registry record in the field “Location” (for ClinicalTrials.gov), “Contact’s address” (for ISRCTN.org and Netherlands National Trial), and “Contact person” (for Australian New Zealand Clinical Trials Registry). We considered that the number was not reported if we could not find at least one result in the fields mentioned above. We classified the trial “unclear multicentric” if the term multicentric was used to describe the trial with no details on the centers involved.

### Intervention

We recorded the type of intervention (for example, programmed interaction with a therapist, Web-based program, use of educational material (DVD, booklet) or other type of intervention), and the involvement of other people closely related to the patient in the educational intervention.

We assessed whether items related to the intervention were reported according to the recommendations of the Consort Statement extension for trials of non-pharmacologic treatment [8]. We first determined whether qualitative data were reported and whether the record included information on the mode of delivery (for example, group or individual), care providers and how the intervention was standardized. We determined whether the content of educational sessions was 1) clearly described (that is, all key components reported), 2) available in a publication or on a website referenced in the record, or 3) not reported (that is, many important pieces of information were missing).

We assessed the reporting of quantitative data, such as the number, frequency and duration of sessions and the course period (that is, the period when participants received the intervention).

If several interventions were assessed, we reported the best-described intervention in the data extraction form.

### Statistical analysis

We used descriptive statistics for continuous variables: median [interquartile range], minimum and maximum values. Categorical variables were described with frequencies and percentages. Data analyses involved use of R for Windows, release 2. 9.

## Results

### Studies selected

**Figure 1:**
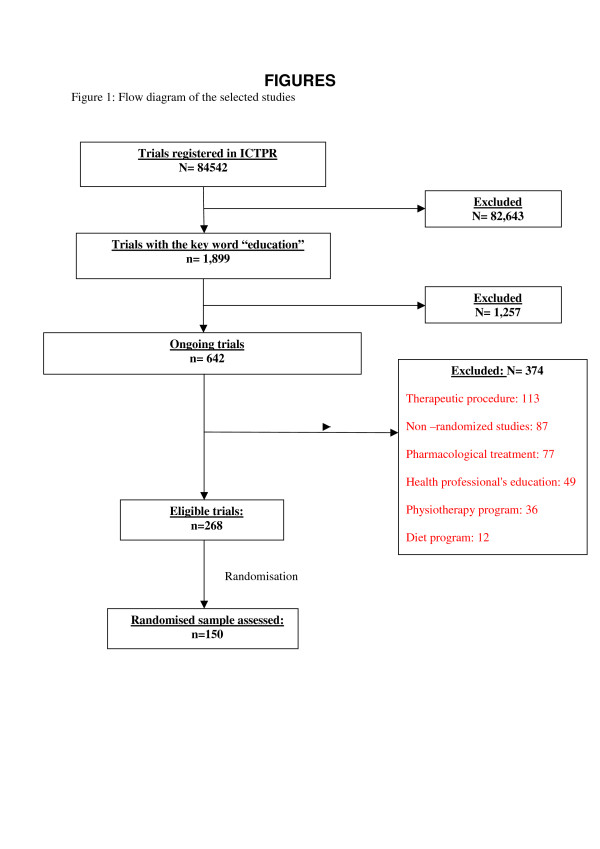
Flow diagram of the selected studies.

Figure 1 describes the trial selection process. We selected 268 of 642 potentially eligible ongoing studies, then randomly selected and appraised 150 studies (56% of the eligible sample).

### Participants and setting

All selected trials were registered in four registers, mainly ClinicalTrials.gov (60.7%). The median target sample size was 205 [100 to 400] patients. The comparator(s) were mainly usual care (47%) or an active treatment (47%). Of these, most concerned other educational interventions [14].

Study characteristics are summarized in Table 1. In total, 70 trials (47%) were taking place in the United States and 38 (26%) in Europe; no study involved low-income countries. When centres were reported, most trials (64%) involved only one centre. Participating centres were mainly tertiary-care, academic or university hospitals (51%).

**Table 1:** Study characteristics

**Items**	**N = 150 n (%)**
**Type of patients**^1^	
Healthy	42 (28.0)
Sick	110 (73.3)
**Country/Continent**	
United States	70 (46.7)
Europe	38 (25.4)
United Kingdom	10 (26.3)
France	1 (2.6)
Australia	21 (14.0)
Canada	13 (8.7)
Asia	4 (2.7)
Africa	3 (2.0)
South America	1 (0.7)
**Number of centres**	
Monocentric	96 (64.0)
Multicentric (reporting number)	35 (23.3)
Unclear multicentric	13 (8.7)
Not reported	6 (4.0)
**Type of centre**^**1**^	
Tertiary care/academic/university hospital	77 (51.3)
General medical hospital	29 (19.3)
Physician/pharmacist/primary care setting	3 (2.0)
Unclear	3 (2.0)
Not reported Other	6 (4.0)47 (31.3)
**Mode of recruitment**^**1**^	
Academic/university hospital	17 (11.3)
“Direct” recruitment from a general population	14 (9.3)
General medical hospital Other	13 (8.7)17 (11.3)
Physicians/pharmacist/primary care setting	8 (5.3)
Unclear	4 (2.7)
Not reported	86 (57.3)

^1^Multiple answers were possible, so the total does not equal 100%.

### Intervention

Most studies (84%) used programmed interaction (that is, education sessions or phone calls); 30% used educational material (that is, DVD or booklet) and 11% an Internet program (Table 2). Of 150 interventions studies, 38 (25%) involved other people closely related to the participant.

**Table 2:** Description of the intervention

**Items**	**N = 150 n (%)**
**Components**^**1**^	
Programmed interaction	126 (84.0)
Internet program	16 (10.7)
Educational material	45 (30.0)
Other	7 (4.7)
Not reported	5 (3.3)
**Involvement of other people closely related to the patient**	38 (25.3)
**Reporting of**	
**Mode of delivery**^**2**^**(n = 126)**	65 (51.6)
**Number of sessions/calls/contact**^**2**^**(n = 134)**	100 (74.2)
**Frequency of sessions/calls/contact**^**2**^**(n = 134)**	78 (58.2)
**Duration of each sessions/calls/contact**^**2**^**(n = 137)**	61 (44.5)
**Overall duration of the intervention**	94 (62.7)
**Description of the content of the intervention**	
Reporting all the key components	45 (30.0)
Content referenced and available	17 (11.3)
Content not reported or much information missing	88 (58.7)
**Reporting of**	
**Information about care provider**^**2**^**(n = 146)**	44 (30.1)
**Methods of standardization**^**2**^**(n = 135)**	9 (6.7)
**Reference(s) to previous publication(s)**	42 (28.0)
**Availability of a website that provides additional data**	
Yes - free and accessible	17 (11.3)
Yes - but not free access	4 (2.7)
Yes - but not accessible	5 (3.3)
No	124 (82.7)

^1^Multiple answers were possible, so the total does not equal 100%.

^2^Percentage of study reports in which the item was applicable (n), that is, interventions involving exclusively an Internet program or educational material were classified “not applicable” for the items reporting the mode of delivery and number, frequency and duration of sessions.

A minority of records (17%, 95% CI 11 to 23%) reported an overall adequate description of the intervention (that is, description that reported the content, mode of delivery, number, frequency, duration of sessions and overall duration of the intervention. For most reports, qualitative data were not reported. For 88 (59%) studies, the content of the educational sessions was not reported. The content was clearly described for 30% (n = 45) of the studies and the description of the content was available in a publication for 28% (n = 42). In total, 11% (n = 17) of reports referenced a website that provided access to educational materials; none of the other 89% of reports provided additional materials.

Information about the care providers administering the intervention was available for 30% (n = 44) of the trials and methods of standardization were specified for 7% (n = 9).

The mode of intervention delivery was reported for 52% (n = 65) of the studies, the number of sessions for 74% (n = 100), the frequency of sessions for 58% (n = 78), the duration of each session for 45% (n = 61) and the overall duration of the intervention for 63% (n = 94) (Tables 3 and 4).

**Table 3:** Some examples of missing and reported elements of interventions

**Intervention's description**	**Reported and missing elements**
*Title: “Arthritis Self-Management Education Program”*	
“Participants will receive two weeks, lay led, workshop focusing on goal setting, problem solving, and content specific to disease management.” [15]	**Reported elements:**overall duration: two weeks **Missing elements:** mode of delivery, number, frequency, duration and content of sessions, care provider's qualification, standardization method
*Title: Evaluation of an Online Fatigue Self-management Group Intervention for Adults with Multiple Sclerosis*	
“This 7-week intervention will follow the published protocol outlined in Managing Fatigue (Packer *et al*., 1995). Full participation requires 2 hours per week of online contribution. Each session is highly structured and includes an education session, practice activities, discussions and homework assignment. The topics include the importance of rest, communication and body mechanics, organizing work stations, setting priorities and standards, balancing schedules and setting goals. All teaching content, worksheets, and homework assignments are provided online. Also participants can share information, express their ideas or feelings and offer advice or support to one another. Facilitators administering the interventions will be qualified health professionals (occupational therapists, nurses or social workers) who have completed a 2-day training course.” [16]	**Reported elements:** Content: quickly described but referenced overall duration: six weeks number: six sessions frequency: weekly duration of sessions: two hours mode of delivery: online standardization methods: highly structured sessions care provider's qualification: occupational therapists, nurses or social workers trained for two days **Missing elements:** none

**Table 4:** Proposals for the reporting of educational intervention

**Domains**	**Examples**
**Describe the access to all intervention materials used**	
· Publicly available access**:** description of modalities of access · Non publicly available access: description of conditions and process of access	Availability of a link to a trial website that contains all materials For intervention materials, contact the investigator Dr. … at the following address …@…
**Describe the intervention materials considered**	
· Materials used by participants	Booklet, leaflets, website, homework assigned
· Materials used by care providers	Training guide, interview guide, phone contact guide, questionnaires, manuals, schedule, online case manager module
· Other materials available describing interventions	Video of session, protocols
**Provide a comprehensive description of the intervention**	
· **The content of the intervention**	
List and describe all components	“The education and support package consists of a written education booklet that provides tailored information, supplemented by verbal reinforcement and repetition of the information contained therein stroke.” [17]“The written education booklet contains topics including the definition, causes, warning signs, risk factors, effects, diagnosis and treatment of stroke, as well as rehabilitation, recovery, returning to activities, going home, practical management strategies and services and support available after stroke.” [17]
· **The modalities of delivery**	
Describe the mode of delivery	“This verbal reinforcement will occur both face-to-face (prior to hospital discharge) and over the telephone (after hospital discharge).” [17]
Describe the course period	“The treatment will run for up to 3 months post-discharge.” [17]
Describe the number, the frequency and the duration of each educationalsession	“Full participation requires 2 hours per week of online contribution for 6 weeks.” [16]
· **The care provider interacting with participants**	
Describe qualifications	“Intervention consists in meets with oncology nurse to have questions answered about medical treatments and side effects.” [18]
Describe level of experience	“The intervention group will receive chemotherapy education from an experienced nurse prior to their first chemotherapy which may last up to one hour.” [19]
Describe the duration of training	“Facilitators administering the interventions will be qualified health professionals who have completed a 2-day training course.” [16]
· **The methods used to standardize interventions and control adherence to protocol**	
Describe methods of standardization	“The online cardiac case manager has a separate content/patient management structure, which allows them to track the progress in terms of readings, educational activities and patient self-report data.” [20]
Describe the methods used to control adherence to protocol	“A proportion of sessions will be taped and evaluated by an independent assessor to ensure that Group CBT strictly follows the manual.” [18]

## Discussion

This study assessed information included in trial registration for a representative random sample of 150 ongoing RCTs assessing an educational intervention, a non-pharmacologic treatment, in the WHO International Clinical Trial Registry Platform. For most trials (59%), interventions were insufficiently described in terms of educational content, modes of delivery and care providers’ qualifications. Most trials (73%) took place in the United States or the United Kingdom, 64% involved only one centre, and participating centres were mainly tertiary-care, academic or university hospitals (51%). The lack of adequate description in such trials raises doubts about the ability of trial registration to help patients and researchers know about the treatment evaluated.

Prospectively registering clinical trials, by placing key protocol information about the trial in the public domain, has been promoted to prevent misconduct in trials, specifically selective reporting [21]. The lack of adequate reporting of the interventions in registries raises several problems for patients, clinicians and researchers. First, knowing precisely the content of an experimental intervention is a preliminary step for patients who wish to participate in an ongoing trial. As well, clinicians and researchers need to have a clear description of interventions that are currently assessed when they plan new trials. Finally, researchers performing meta-analyses need to precisely identify ongoing studies meeting their inclusion criteria.

Our findings may be explained in part by the lack of consideration of the specifics of non-pharmacologic interventions in guidelines for trial registration. For example, guidelines for the item dedicated to the intervention in the WHO Trial Registration Data Set are “*If the intervention consists of several separate treatments, list them all (e.g., "low-fat diet, exercise"). For each intervention, describe other intervention details as applicable (dose, duration, mode of administration, etc.)*”. These recommendations are appropriate for drugs but not for non-pharmacologic treatments. Indeed, educational interventions are complex, involve several components [7] and are, therefore, difficult to describe, standardize, reproduce [8].

Space constraints in both scientific journals and trial registration databases may restrict the full reporting of interventions [22]. Potential solutions to providing complete descriptions include Internet hyperlinks to additional information and educational materials, establishing a stable “intervention bank” where materials and procedures are made available [22] and the use of graphics to depict the flow and timing of sessions of treatment [23].

However, the complete reporting of items in trial registries raises issues related to intellectual property. Research teams that developed these interventions have invested time and money and are entitled to protect their data before the publication of the study results.

Our results are consistent with those of previous studies investigating the quality of reporting in trial registries [21], [24-26]. As well, previous studies demonstrated inadequate descriptions of non-pharmacologic treatment in published articles [11], [27-29]. Glasziou *et al.*[27]. reported that about 70% of reports of trials evaluating non-pharmacologic treatment provided insufficient information on the intervention to allow for replication in practice.

The inferences from this study are limited by the inherent limitations of trial registries. The search strategy may not have identified all studies evaluating educational interventions. Registries have limited advanced search capabilities; contrary to search strategies for databases of published articles, such as MEDLINE or the Cochrane Central Register of Controlled Trials, search strategies of registries have not been evaluated. In addition we did not write to the investigators for clarification.

## Conclusions

We show that educational interventions assessed in ongoing RCTs are poorly described in trial registration. The lack of adequate description raises doubt about the ability of trial registration to help patients and researchers know the treatment evaluated.

## Abbreviations

ICMJE: International Committee of Medical Journal Editors; WHO: World Health Organization; CONSORT: Consolidation of the standards of reporting trials; RCTs: Randomized controlled trials; ICTRP: International Clinical Trials Registry Platform.

## Competing interests

The authors declare that they have no competing interests.

## Authors’ contributions

CP participated in the study concept and design, the literature search and identifying relevant trial records and the acquisition of data from included records; performed the statistical analysis; and participated in the analysis and interpretation of data and drafted the manuscript. IB participated in the study concept and design, the literature search and identifying relevant systematic reviews, the analysis and interpretation of data, the critical revision of the manuscript for important intellectual content and the study supervision. PR participated in the study concept and design, the analysis and interpretation of data and the critical revision of the manuscript for important intellectual content; and supervised the study. CP is guarantor and has full access to all of the data in the study and takes responsibility for the integrity of the data and the accuracy of the data analysis. All authors read and approved the final manuscript.

### Funding

This study was funded by a grant “Recherche infirmière – offre de formation doctorat” of the “Assistance Publique - Hôpitaux de Paris”.
